# A rare case of accessory liver lobe torsion in a pediatric patient who showed recurrent epigastralgia and who was treated by elective laparoscopic resection

**DOI:** 10.1186/s40792-021-01231-6

**Published:** 2021-06-15

**Authors:** Ayaka Nagano, Shun Onishi, Chihiro Kedoin, Mayu Matsui, Masakazu Murakami, Koshiro Sugita, Keisuke Yano, Toshio Harumatsu, Koji Yamada, Waka Yamada, Makoto Matsukubo, Mitsuru Muto, Tatsuru Kaji, Satoshi Ieiri

**Affiliations:** 1 Department of Pediatric Surgery, Research Field in Medical and Health Sciences, Medical and Dental Area, Research and Education Assembly, Kagoshima University, 8-35-1, Sakuragaoka, Kagoshima, 890-8520 Japan; 2 Clinical Training Center, Kagoshima University Hospital, Kagoshima, Japan

**Keywords:** Accessory liver lobe, Torsion, Epigastralgia, Laparoscopic surgery, Children

## Abstract

**Background:**

Accessory liver lobe (ALL) is a rare liver malformation. An ALL develops due to malformation of the endodermal caudal foregut and segmentation of the hepatic bud in the third week of gestation. Most ALLs are asymptomatic and are detected incidentally during abdominal surgery. The incidence of ALL is < 1% in patients who undergo abdominal surgery. However, some ALLs twist and cause acute abdomen. We experienced a pediatric case of ALL torsion in a patient who underwent elective laparoscopic surgery.

**Case presentation:**

The 5-year-old girl had a 3-month history of epigastralgia and vomiting, which occurred every 2 weeks. Abdominal ultrasonography with color Doppler imaging revealed an 11.8 × 13.6 mm nonvascular lesion with mixed echogenicity near the round ligament of the liver. Enhanced computed tomography confirmed a 14 × 16 × 20 mm low-attenuation mass surrounded by a hyperdense line and disproportionate fat stranding on the right side of the round ligament of the liver. There was no ascites or hemorrhage. These findings suggested an abscess of the round ligament of the liver. Her symptoms improved with the administration of oral antibiotics; thus, we planned to perform elective exploratory laparoscopy and subsequent resection. Two trocars (5 mm) were inserted through a multichannel port device at the umbilicus and one trocar (3 mm) was inserted at the right lateral abdomen. Upon observation of the abdominal cavity, the omentum was observed adhering to the round ligament of the liver. Macroscopic observation revealed no apparent mass lesions. We performed adhesiolysis of the omentum from the round ligament of the liver using a vessel sealing system. We performed resection at the site at which adhesion had formed between the round ligament of the liver with the surrounding tissue using a vessel sealing system and the resected specimen was extracted through the umbilical wound. The postoperative course was uneventful. A pathological examination revealed necrotic liver tissue. The resected tissue was founded to be an ALL with ischemic change.

**Conclusions:**

The recurrent abdominal pain was induced by torsion of the ALL. Pediatric surgeons should consider ALL torsion as a differential diagnosis for epigastralgia of unknown etiology.

**Supplementary Information:**

The online version contains supplementary material available at 10.1186/s40792-021-01231-6.

## Background

Accessory liver lobe (ALL) is a rare liver malformation. An ALL develops as a result of the malformation of the endodermal caudal foregut and segmentation of the hepatic bud in the third week of gestation [1]. Most ALLs are asymptomatic, with some detected incidentally during abdominal surgery. The incidence of the ALL was reported to be < 1% in patients undergoing laparoscopic surgery [2]. However, in some cases, torsion of the ALL occurs, causing acute abdomen. Twenty-four pediatric cases of ALL torsion have been reported [3–5]; most were resected in an emergency operation. We herein report a rare case of ALL torsion in a pediatric patient who showed recurrent epigastralgia, and who was treated by elective laparoscopic resection.

## Case presentation

A 5-year-old girl with history of mackerel allergy, atopic dermatitis and chronic constipation, had a 3-month history of epigastralgia and vomiting, which occurred repeatedly, every 2 weeks. A pediatrician performed physical and laboratory examinations when she had no symptoms; however, no abnormal findings were recognized. Four months later, she was transferred to our institution for a detailed workup because of recurrent epigastralgia.

**Fig. 1:**
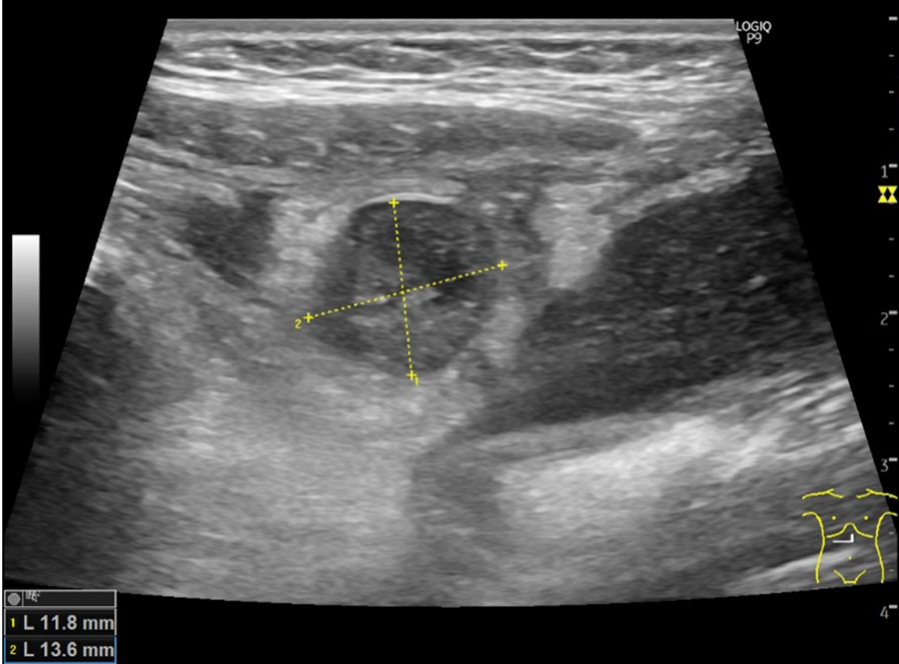
Abdominal ultrasonography with color Doppler imaging. A nonvascular lesion of mixed echogenicity was recognized near the round ligament of the liver

**Fig. 2:**
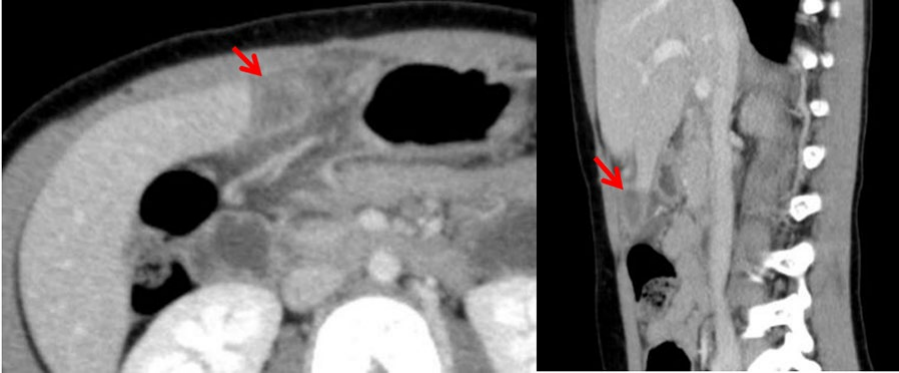
Enhanced computed tomography scan of the abdomen. A low-attenuation mass surrounded by a hyperdense line and disproportionate fat stranding on the right side of the round ligament of the liver

On abdominal examination, a small palpable mass was identified in the upper abdomen. A blood analysis revealed the following: white blood cell count, 10,100 /μl; hemoglobin, 13.2 g/dl; and C-reactive protein, 0.66 mg/dl. The patient’s liver enzyme levels were within the normal ranges. On abdominal ultrasonography with color Doppler imaging, an 11.8 × 13.6 mm nonvascular lesion with mixed echogenicity was detected near the round ligament of the liver (Fig. 1). Enhanced computed tomography (CT) confirmed a 14 × 16 × 20 mm mass with low attenuation surrounded by a hyperdense line with disproportionate fat stranding on the right side of the round ligament of the liver (Fig. 2). There was no ascites or hemorrhage in the abdominal cavity. These findings suggested an abscess of the round ligament of the liver. Her symptoms were improving; thus, we prescribed oral antibiotics and planned to perform elective laparoscopic exploration followed by resection. After her last admission to the hospital, she had no symptoms for approximately 2 months until she underwent surgery. The target lesion was small and was detected preoperatively. We therefore decided to perform two-site laparoscopic surgery [6].

**Fig. 3:**
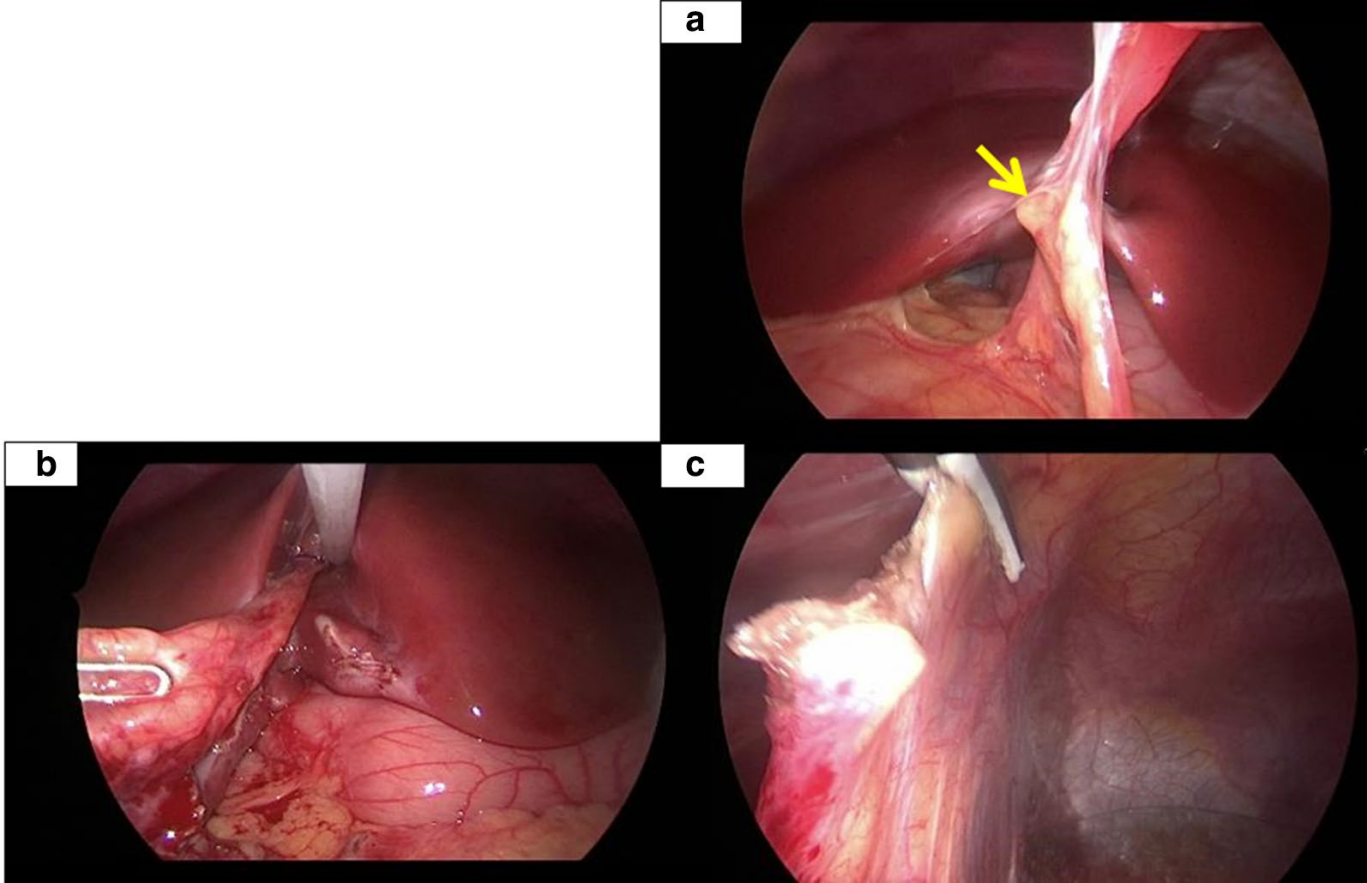
Operative findings. **a** The omentum was adherent to the round ligament of the liver. The arrow points the ALL. **b** Resecting adhesion from around the round ligament of the liver. **c** Ligation of the round ligament of the liver attached to the liver

Under general anesthesia, the patient was placed in the broard base position. The umbilicus was opened through a 1.5 cm longitudinal incision, and a wound retractor (LAP PROTECTOR Mini-mini; Hakko Co. Ltd, Tokyo, Japan) was applied. Two 5-mm trocars (scope and operator’s right hand, respectively) were inserted through multichannel port device (E・Z access; Hakko Co. Ltd) to attach to the wound retractor and pneumoperitoneum was established (8 mm Hg, 5 L/min CO_2_ insufflation). In addition, a 3-mm trocar (operator’s left hand) was inserted at the right lateral abdomen under a laparoscopic view (30° rigid scope, KARLSTORZ, Tuttlingen, Germany). Observation of the abdominal cavity revealed adhesion between the omentum and the round ligament of the liver (Fig. 3a). Macroscopic observation revealed no apparent mass lesions. We performed adhesiolysis of the omentum from the round ligament of the liver using a vessel sealing system (LigaSure™ Maryland; Medtronic, Dublin, Ireland) (Fig. 3b). After ligation of the round ligament of the liver attached to the liver site using a loop suture (ENDOLOOP® Ligature; Johnson & Johnson, NJ, USA) (Fig. 3c), we resected the round ligament of the liver with the surrounding tissue using a vessel sealing system (Additional file 1: Video). The resected specimen was extracted through the umbilical wound. The postoperative course was uneventful. She was discharged from hospital 3 days after surgery and remained asymptomatic for approximately half a year.

**Fig. 4:**
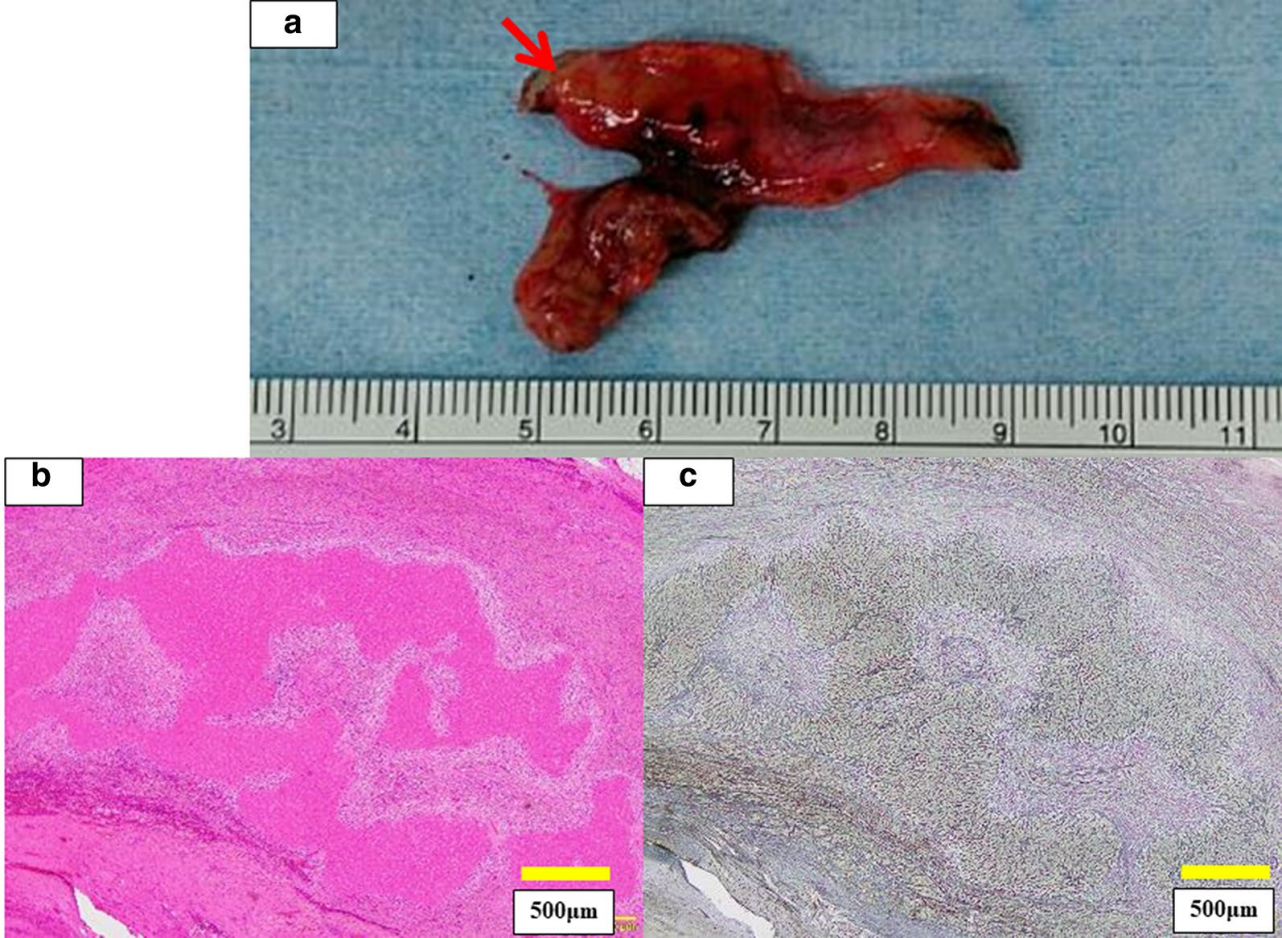
Pathological findings. **a** Surgical resection specimen. The arrow points to the ALL. **b** Hematoxylin–eosin staining. **c** Silver impregnation staining

The resected specimen is shown in Fig. 4a. Microscopically, 6 mm of granulomatous tissue was observed around the round ligament. The collection of multinucleated giant cells and histiocytes, hemosiderin deposition and an area of coagulative necrosis with lymphocyte infiltration were also detected (Fig. 4b). Necrotic liver tissue in the area of necrosis was detected by silver impregnation staining (Fig. 4c). We concluded that the resected tissue was an ALL with ischemic change.

## Discussion

Liver malformations include ALL and ectopic liver. Different definitions and classifications are applied. The most widely applied classifications categorize liver malformation into four types: (1) large ALL (> 30 g) attached to the liver by a stalk; (2) small ALL (≤ 30 g) attached to the liver; (3) ectopic accessory liver without any connection to the liver; and (4) microscopic ectopic liver [3, 7]. Types 1 and 2 are classified as ALL, which has the potential for torsion, while types 3 and 4 are classified as ectopic liver, which does not have the potential for torsion.

An ALL may prevent fusion of the anterior abdominal wall and is frequently associated with anterior abdominal wall defects [1]. Corbitt et al. reported that, 41 % of 22 children with ALL showed associated abdominal wall defects, including omphalocele, Beckwith–Wiedemann syndrome, umbilical hernia and cloacal exstrophy [3].

The exact cause of ALL torsion is often unknown, but some studies reported that it might be caused by strenuous exercise or trauma [8]. After torsion, ALL sometimes shows spontaneous resolution. However ALL torsion has the potential to cause infarct, rupture, fracture, bleeding, hemangioma, biliary obstruction and gallbladder torsion. They potentially cause symptoms, such as acute or recurrent abdominal pain, nausea, vomiting, anorexia, pallor or cyanosis due to hypovolemic shock, impairment of the liver function or cholestasis [4, 9]. Most reported cases follow an acute course. Few cases showed recurrent symptoms and only one adult case was treated by elective resection [10]. In our case, the patient’s 3-month history of recurrent epigastralgia and vomiting suggested that the ALL may have been repeatedly twisted and spontaneously resolved. Finally, the ALL was completely twisted; however, her symptoms disappeared spontaneously. We hypothesize that her symptoms disappeared because the ALL was small and as a result, ALL became completely necrotic.

Ultrasonography, non-contrasted or enhanced CT and magnetic resonance imaging (MRI) are used for the diagnosis of ALL. However, most cases were not diagnosed preoperatively [11, 12]. In our case, on ultrasonography and CT, the twisted ALL appeared as an ischemic or congested mass lesion. On enhanced CT, a low-attenuation mass surrounded by a hyperdense line and disproportionate fat stranding raised suspicion of an abscess; however, the hyperdense line on non-enhanced CT suggested hemorrhagic necrosis due to torsion of an ALL. In addition, it was reported that in some cases, adjacent fat stranding may occur in the presence of surrounding inflammation due to torsion [3, 11]; thus, the disproportionate fat standing in our case might have been due to torsion of the ALL. MRI is also reported to be more sensitive than CT for obtaining a definitive diagnosis [12]; thus, it may be useful for a differential diagnosis in non-emergent cases.

The surgical indications for asymptomatic ALL are controversial. Generally, cases of asymptomatic ALL are observed. However, torsion of a large ALL with strangulation of the vascular supply to the liver may potentially lead to severe hepatic ischemia. Ladurner et al. reported a case of torsion of a large ALL in which liver transplantation was required [13]. A large ALL with a stalk should be resected before torsion occurs. However, torsion of small ALLs has also reported [14]. The size of the ALL might be not necessarily proportional to the ease of torsion. In cases in which ALL torsion is suspected, prompt management and emergency exploratory laparotomy or laparoscopy should be recommended in order to avoid a potentially life-treating condition. Additionally, there are cases that was suspected tumor or abscess by imaging [1]. If we cannot rule out these possibilities, we consider that we should perform surgery for diagnosis.

Laparoscopic resection of ALL torsion has been reported in pediatric cases [3, 11]. There are no reports of the resection of an ALL located near the round ligament of the liver. The laparoscopic resection of an abscess of the falciform ligament has been reported [15]. According to this report, a surgeon should use vessel clips or an ultrasonically activated device to prevent hemorrhage from the branches of the internal thoracic vein or the inferior phrenic vein. After torsion is established, elective surgery may be allowed depending on the general condition and symptoms of the patient. The performance of 2-site laparoscopic extirpation allows excellent cosmetic outcomes. We previously reported that even trainee could perform a 2-site laparoscopic appendectomy safely [6]. Our approach is also feasible for elective surgery of ALL.

## Conclusions

We successfully treated a pediatric patient with ALL torsion. A definitive diagnosis of small ALL is difficult to make before surgery. However, laparoscopic elective resection is acceptable for a pediatric patient with a small ALL with no symptoms. Pediatric surgeons include ALL torsion in the differential diagnosis epigastralgia of unknown etiology.

## Supplementary Information

**Additional file 1.** Video.

## Data Availability

The data used in this report are available from the corresponding author on request.

## Abbreviations

ALL: Accessory liver lobe
CT: Computed tomography
MRI: Magnetic resonance imaging

## References

[1] Al-Anwar S, Askari A, Alvi A (2019). Right Iliac Fossa Pain due to torsion and ischemia of accessory liver lobe. J Surg Case Rep.

[2] Sato S, Watanabe M, Nagasawa S, Niigaki M, Sakai S, Akagi S (1998). Laparoscopic observations of congenital anomalies of the liver. Gastrointest Endosc.

[3] Corbitt N, Rellinger EJ, Hernanz-Schulman M, Chung DH (2017). Accessory hepatic lobes in the pediatric population; A report of three cases of torsion and literature review. J Pediatr Surg Case Rep.

[4] Thakral CK, Shivalingam G, Dar FM, Thakral N (2017). Torsion of an accessory hepatic lobe with embedded gallbladder: in an 11-month-old boy. European J Pediatr Surg Rep.

[5] Calame P, Lenoir M, Delabrousse E (2021). Imaging features of accessory liver lobe torsion. Diagn Interv Imaging.

[6] Moriguchi T, Machigashira S, Sugita K, Kawano M, Yano K, Onishi S (2019). A randomized trial to compare the conventional three-port laparoscopic appendectomy procedure to single-incision and one-puncture procedure that was safe and feasible, even for surgeons in training. J Laparoendosc Adv Surg Tech A.

[7] Collan Y, Hakkiluoto A, Hästbacka J (1978). Ectopic liver. Ann Chir Cynaecol.

[8] Koplewitz BZ, Manson DE, Ein SH (1999). Posttraumatic torsion of accessory lobe of the liver and the gallbladder. Pediatr Radiol.

[9] Wang C, Cheng L, Zhang Z, Xie T, Ding H, Deng Q (2012). Accessory lobes of the liver: a report of 3 cases and review of the literature. Intractable Rare Dis Res.

[10] Jambhekar K, Pandey T, Kaushik C, Shah HR (2010). Intermittent torsion of accessory hepatic lobe: an unusual cause of recurrent right upper quadrant pain. Indian J Radiol Imaging.

[11] Salisbury SM, Yi CE, Merianos DJ, Sapra A, Anselmo DM (2013). Laparoscopic resection of a torsed accessory hepatic lobe: case report and literature review. J Pediatr Surg Case Rep.

[12] Khan AM, Hundal R, Manzoor K, Dhuper S, Korsten MA (2006). Accessory liver lobes: a diagnostic and therapeutic challenge of their torsions. Scand J Gastroenterol.

[13] Ladurner R, Brandacher G, Mark W, Iannetti C, Lottersberger C, Steurer W (2005). Complete hepatic ischemia due to torsion of a large accessory liver lobe: first case to require transplantation. Transpl Int.

[14] Kawaguchi Y, Mitsunaga T, Saito T, Terui K, Nakata M, Yoshida H (2018). Case report: torsion of an accessory liver lobe in a 6-year-old girl. J Jpn Soc Pediatr Surg..

[15] Czymek R, Bouchard R, Hollmann S, Kagel C, Frank A, Bruch HP (2010). First complete laparoscopic resection of a gangrenous falciform ligament. Eur J Gastroenterol Hepatol.

